# Remote mental health clients prefer face-to-face consultations to telehealth during and after the COVID-19 pandemic

**DOI:** 10.1177/10398562211043509

**Published:** 2022-02

**Authors:** Andrew James Amos, Jocelyn Middleton, Fergus W. Gardiner

**Affiliations:** Director of Training, Psychiatry for North Queensland, Townsville, QLD; Manager, Mental Health and Wellbeing teams, Royal Flying Doctor Service, Cairns, QLD; Director, Public Health and Research, Royal Flying Doctor Service, Federation, Canberra, ACT

**Keywords:** rural mental health, telehealth, Indigenous psychiatry, administrative mental health

## Abstract

**Objective:**

To guide the efficient and effective provision of mental health services to clients in Central West and Far North Queensland, we surveyed preferences for face-to-face or in-person contact.

**Methods:**

A clinician-designed survey of contact preferences was offered to 248 clients of mental health services in Far North and Central West Queensland in mid-2020. With the onset of COVID-19, the survey was modified to measure the impact of the pandemic.

**Results:**

Just over half of the services’ clients participated in the survey (50.4%), of whom more were female (63.2%). Of the participants, 46.3% in Far North and 8.6% in Central West Queensland identified as Indigenous. Strong resistance to telehealth before the pandemic across groups (76%) was moderated during COVID-19 (42.4%), an effect that appeared likely to continue past the pandemic for Central West clients (34.5%). Far North clients indicated their telehealth reluctance would return after the pandemic (77.6%).

**Conclusions:**

Our results suggest that remote Australians strongly prefer in-person mental health care to telehealth. Although the COVID-19 pandemic increased acceptance of telehealth across regions while social distancing continued, there was evidence that Indigenous Australians were more likely to prefer in-person contact after the pandemic.

Rural and remote Australia has higher rates of chronic disease and completed suicide than metropolitan areas, with lower healthcare provision.^1^ The COVID-19 pandemic has exacerbated mental health disparities, with increased emergency retrieval from remote areas for mental and behavioural conditions.^2^ Lower mental health service provision^3^ appears to be a factor, with rural mental health clients required to travel long distances, wait for travelling clinics, or forgo care altogether.^2^

Rural and remote services have long relied on telehealth,^4^ and social distancing during the COVID-19 pandemic saw its significant expansion across metropolitan services.^5^ While some articles have considered perceptions of telehealth during COVID-19,^6^ there is limited research on mental health client preferences for face-to-face or telehealth consultations.

In 2019, to guide service development, we designed a survey of remote mental health clients’ preference for telehealth or face-to-face contact. On the emergence of COVID-19 in 2020, we modified the survey to explore whether the pandemic had influenced immediate and future contact preferences.

## Methods

This project was deemed a low-risk quality assurance project by the Far North Queensland Human Research Ethics Committee (review number 1499 AB).

### Setting

This study surveyed Queensland clients accessing remote mental health services operated by the Royal Flying Doctor Service (RFDS) in Far North and Central West Queensland (FNS/CWS). Both services comprise mental health nurses, social workers, occupational therapists and psychologists providing culturally appropriate, evidence-based, psychological therapies to individuals who present with mild to moderate mental health needs.

Remote Australian communities have distinctly different service accessibility and disease outcomes. The FNS and CWS are representative of the diverse populations supported by the RFDS, including a higher proportion of Indigenous clients in FNS than CWS.

### Sample design and recruitment

All current adult clients (>17 years old) of FNS and CWS in RFDS’ electronic medical record were included in the sample.

### Exclusion criteria

Individuals in hospitals, nursing homes, prisons, hotels and hostels at the time of the survey, and visitors usually residing outside Australia, were excluded.

### Survey procedures

Three attempts were made to contact participants by phone by four FNS/CWS clinicians. Respondents were asked to complete a survey, with verbal consent obtained from each participant to record, collate, and present their answers in summary form.

### Measures

Consenting clients were asked questions created by consensus among senior clinicians (including JM). Answers were recorded in text and responses coded by interviewers (Table 1).

**Table 1.** Participant survey questions

***Participants to indicate one option only.*** Prior to the pandemic: a. Did you prefer face to face sessions with the RFDS clinician? b. Did you prefer telehealth sessions with the RFDS clinician? c. Were you equally happy with either face-to-face or telehealth sessions with the RFDS clinician?
***Participants to indicate one option only.*** As a result of the COVID-19 pandemic: a. Would you prefer telehealth only during pandemic periods? b. Would you prefer your telehealth consultations to continue beyond pandemic periods? c. Are you equally happy with either face-to-face or telehealth consultations beyond pandemic periods?

### Sample

Between May and June 2020, attempts were made to contact 248 clients, with a final sample of 125 participants (50.4%). Of 123 non-participants, 25 had no recorded phone number, 93 did not respond after three attempts, and six declined.

Non-participation was higher in FNS (*n* = 81; 54.7%) than CWS (*n* = 42; 42.0%) partly because more clients had no recorded phone number (*n* = 23; 15.5% vs *n* = 2; 2.0%).

## Statistical analysis

Continuous variables were summarised as medians and interquartile ranges (IQR). Categorical variables were summarised as counts and proportions. We used chi-square goodness of fit to compare proportions with a significance threshold of 0.05.

Statistical analyses were performed using the statistical software package R version 3.5.1. (R Foundation for Statistical Computing, Vienna, Austria).

## Results

Participant characteristics are summarised in Table 2.

**Table 2.** Characteristics of survey participants

Description	Far North (%)	Central West (%)	Total (%)
**Total number of clients contacted for survey participation**	148	100	248 (64.1)*
**Clients who participated in the survey**	67 (45.3)	58 (58.0)	125 (50.4)
**Clients who did not engage with the survey**	81 (54.7)	42 (42.0)	123 (49.6)
Number of clients not able to be reached via phone	23 (15.5)	2 (2.0)	25 (10.1)
Number of clients who did not answer	54 (36.5)	39 (39.0)	93 (37.5)
Number of clients who declined to participate	4 (2.7)	1 (1.0)	5 (2.0)
**Participating clients identifying as Indigenous Australians**	31 (46.3)	5 (8.6)	36 (14.5)
**Participant gender**			
Number of participants who identified as male	24 (35.8)	22 (37.9)	46 (36.8)
Number of participants who identified as female	43 (64.2)	36 (62.1)	79 (63.2)
**Participant age in years**			
Number aged 18–34	27 (40.3)	20 (34.5)	47 (37.6)
Number aged 35–44	10 (14.9)	11 (19.0)	21 (16.8)
Number aged 45–54 (%)	16 (23.9)	13 (22.4)	29 (23.2)
Number aged 55–64 (%)	10 (14.9)	11 (19.0)	21 (16.8)
Number aged 65+ (%)	3 (4.5)	3 (5.2)	6 (4.8)
Median age (interquartile range)	41.1 (28.9-50.7)	43.3 (30.8-53.9)	(29.6-51.2)

*Note*. *During the study period there was 387 total clients, including 229 in the FNS and 158 in the CWS.

There was a strong preference for face-to-face contact during non-pandemic periods (*n* = 95; 76.0%) with no differences (*p* > .05) between FNS and CWS clients. COVID-19 affected preferences, with 44 clients (35.2%) preferring telehealth to face-to-face consultations during the pandemic, and 53 (42.4%) accepting telehealth after the pandemic. CWS clients were significantly (*p* < .05) more likely to report greater acceptance of telehealth after the pandemic (*n* = 38; 65.5%) than FNS clients (*n* = 15; 22.4%) (Table 3).

**Table 3.** Participating client preferences for face-to-face or telehealth during pandemic and non-pandemic periods

Total participant preferences			
Description	Far North	Central West	Total
**General telehealth preference (Non-pandemic)**			
*Number who prefer face-to-face (%)*	52 (77.6)	43 (74.1)	95 (76.0)
*Number who prefer telehealth (%)*	1 (1.5)	1 (1.7)	2 (1.6)
*Number who have no preference (%)*	14 (20.9)	14 (24.1)	28 (22.4)
***Telehealth preference due to COVID-19 pandemic***			
*Number who prefer telehealth only during COVID-19*	24 (35.8)	20 (34.5)	44 (35.2)
*Number who prefer telehealth beyond COVID-19*	15 (22.4)	38 (65.5)	53 (42.4)
*Number who have no preference beyond COVID-19*	28 (41.8)	0 (0.0)	28 (22.4)
Indigenous and non-Indigenous participate preferences			
Description	Indigenous	Non-Indigenous	Total
*Participants (%)*			
***General telehealth preference (Non-pandemic)***			
*Number who prefer face-to-face*	30 (83.3)	65 (73.0)	95 (76.0)
*Number who prefer telehealth*	0 (0.0)	2 (2.2)	2 (1.6)
*Number who have no preference*	6 (16.7)	22 (24.7)	28 (22.4)
***Telehealth preference due to COVID-19 pandemic***			
*Number who prefer telehealth only during COVID-19*	19 (52.8)	25 (28.1)	44 (35.2)
*Number who prefer telehealth beyond COVID-19*	8 (15.1)	45 (50.6)	53 (42.4)
*Number who have no preference beyond COVID-19*	9 (25.0)	19 (21.35)	28 (22.4)

Thirty-six (28.8%) participants identified as Indigenous, and 89 (71.2%) did not, with significantly (*p* < .05) higher identification in the FNS (*n* = 31; 46.3% vs *n* = 5; 8.6%). Both groups preferred face-to-face services absent a pandemic. A higher proportion (*p* < .05) of Indigenous clients preferred that consultations occur via telehealth only during the pandemic (*n* = 19; 52.8% vs *n* = 25; 28.1%). A significantly (*p* < .05) lower proportion of Indigenous clients wanted telehealth beyond the COVID-19 pandemic (*n* = 8; 15.1% vs *n* = 45; 50.6%; Table 3).

## Discussion

Survey respondents were mostly female, with fewer Indigenous Australians and fewer older adults than expected based on the demographic characteristics of the catchment areas.^7^ While our study cannot explain all these differences, the gender and age imbalances are similar to the demographic spread of CWS and FNS clients. The lower than expected proportion of Indigenous Australians may be consistent with evidence of lower levels of engagement with health and government services with complex multifactorial associations,^8^ and is a priority for further research.

The rapid expansion of telehealth services in response to the COVID-19 pandemic in Australia has stimulated reappraisal of the potential benefits and barriers of telehealth for different client groups and populations.^9-10^ Our survey suggests remote populations in Central West and Far North Queensland overwhelmingly prefer face-to-face mental health services, consistent with general populations.^11^ Understandably, most clients strongly preferred to talk in-person with a trusted clinician with whom they have an established relationship.

Lower FNS participation suggests Far North Queensland clients more generally face multiple barriers to engagement which might include cultural, social, and material factors. In this context, it is interesting that Indigenous people’s resistance to telehealth faded during the pandemic, but was predicted to return after the pandemic has resolved. We hypothesise that the pandemic preference reflects strong efforts to protect remote communities from COVID-19 exposure. Greater telehealth resistance in FNS than CWS after the pandemic may result from different patterns of cost and benefit. Communication and cultural factors can prevent (or, done well, facilitate) engagement for Indigenous people.^8^ While these speculations require substantiation, they are consistent with evidence that culturally sensitive and appropriately staffed telehealth services can improve engagement and outcomes for Indigenous Australians.^12^

Thus, both FNS and CWS clients preferred telehealth in some situations, particularly with elevated in-person risks; other research has found greater tolerance for routine appointments where telehealth avoids significant travel.^13^ Most clients strongly preferred in-person contact for high-impact care such as initial assessment, particularly in crisis, or care transitions. Telehealth use during the pandemic may reduce resistance for CWS clients after the pandemic. These results highlight the importance of research suggesting that telehealth may increase equitable access to care in rural and remote Australia with sufficient attention to demographics, stage and severity of illness, social supports, perceived efficacy, and technological factors such as service reliability.^13^ It appears likely that greater resources will be needed the more remote the client, to overcome barriers like limited familiarity with/access to technology and less opportunity to develop trusting relationships with clinicians. Training and models of care appear vital in preparing and engaging clinical staff in effective provision of telehealth services.^14^

Finally, we recommend that close monitoring of the uptake of telehealth in rural and remote Australia be used to strategically address gaps in service provision. While COVID-19 may have reduced telehealth resistance for some clients,^10^ our results and previous research suggest specific groups may continue to face barriers to access.^15^ Pending evidence that telehealth alone or in combination is as effective as in-person care, we recommend that reliance on telehealth be treated as a key indicator of overall gaps in care for remote populations.

The main limitations associated with this study included a non-validated survey created for service development; service providers collecting responses; and a high non-participation rate with non-responders likely to differ from responders, potentially leading to bias.

## Conclusions

Provision of mental health services in remote Australia differs dramatically to services delivered in major city areas, demanding tailored and culturally appropriate services. Recognising the need to balance efficiency and effectiveness for our diverse client populations, we sought to determine whether face-to-face or telehealth provision was preferred by our clients in remote Queensland. Our results suggest that Indigenous Australians were more motivated to maintain social distancing through telehealth during the pandemic, but also more likely to resist telehealth after its resolution. This pattern indicates that there may be an opportunity to use telehealth to improve access to mental health care in rural and remote Australian regions, but realising these benefits will require a systematic approach involving engagement, training, and close monitoring.
